# A study on the relationships between playfulness, physical self-efficacy, and school happiness among middle school students participating in “0th-period physical education class” in South Korea

**DOI:** 10.3389/fpubh.2023.1232508

**Published:** 2023-09-06

**Authors:** Byungchan Lee, Kihong Joung, Wonjae Jeon

**Affiliations:** ^1^Department of Physical Education, Sichuan Agricultural University, Ya'an, Sichuan, China; ^2^Department of Physical Education, Kangnam University, Yongin, Republic of Korea; ^3^Department of Physical Education, Korea National University of Education, Gangnae-myeon, Chungbuk, Republic of Korea

**Keywords:** Korean P.E. system, early morning exercise, early morning physical activity, 0th-period physical education class, 0th-period physical activity, education for happiness

## Abstract

The purpose of this study is to create a scientific basis for the establishment of “0th-period physical education class” activities in schools in the future, with the expectation that the associations of morning exercise can be activated in the Korean educational community. To achieve this goal, the present study aimed to determine the relationship between the playfulness experienced during the early morning exercise of middle school students and their physical self-efficacy and education for happiness. To examine the model, questionnaires were collected from 296 middle school students located in Seoul and Gyeonggi-do, South Korea. Correlation analysis and standard multiple regression analysis were performed to analyze the data using the SPSS 21.0. The findings were as follows: First, the playfulness of the middle school “0th-period physical education class” had a significant effect on physical self-efficacy. Second, playfulness had a significant effect on education for happiness. Thirdly, physical self-efficacy was found to have a significant effect on education for happiness. Based on the results of this study, we suggest that a “0th-period physical education class” with various activities should be held during the legally required time in South Korea.

## 1. Introduction

In South Korea in 2010, the broadcasting of “0th-period physical education class (0th-period PE class)” became a social issue based on research results that showed exercise had a positive effect on the academic brain area and had a significant impact on students’ academic ability (1). As these research results received media attention, the “0th-period PE class” implementation activities began to expand, centering on local offices of education and elementary, middle, and high schools in Korea.

In South Korea, “0th-period PE class” is academically defined as participating in various sports in the morning hours before the start of the regular curriculum (2). In the Korean academic world, the term is used as “Physical Education Class in Period 0,” “Morning Exercise,” and “Physical Education in Period 0 (3),” whereas in other countries, it is known by terms such as “early morning physical activity,”’ before-school physical activity program,” and “early morning exercise” (4–6).

For the past decade or so, “0th-period PE class” has been conducted by teachers and students together during morning self-study or before the start of the first period. The program involves various physical activities, such as walking, soccer, basketball, and circuit training of moderate to high intensity, and is conducted independently by physical education teachers in elementary, middle, and high schools (7). Although “0th-period PE class” has not been implemented as a formal curriculum so far, it is necessary to generate a variety of positive academic evidence on the benefits of early morning physical activity for Korean adolescents in the future to generate positive implications for the growth of the Korean physical education system (5, 8, 9).

Changes and maturity in the middle school period are acquired based on positive activity experiences and can be naturally acquired in emotional activities related to interest and fun. This naturalness can be found in playfulness through sports (10). Generally, playfulness in sports is related to basic human needs, and the need is to perform various functions in human life (11). From this principle, playfulness in the middle school period is related to sociality among human development tasks. This is because there is a strong desire to value relationships with peer groups. Additionally, children and adolescents encounter natural play through group activities, and the peer relationship is closer than that of parents and teachers (12). Playfulness is the subjective experience of an individual in a state of play, or a characteristic mechanism that synchronizes an individual in a state of play. In other words, playfulness is a personal trait (13). Accordingly, it was reported that adolescents who show high playfulness not only have excellent creativity and problem-solving ability, but are also more likely to achieve excellent academic results, and to lead a smooth school life pattern and a high sense of school happiness. One important characteristic of playfulness is deeply related to not accepting various experiences of the environment to which an individual belongs as stress. Therefore, middle school students’ experience of playfulness has the function of promoting individual success necessary for promoting sociality and leadership (14). In other words, playfulness in school physical education classes and activities includes emotional aspects related to fun and sensory physical vitality such as excitement and an individual’s unique emotional leisure that can be immersed in fun and pleasure for a long time or distinguished from others. These factors are important psychological skills to relieve daily stress or improve a close intimacy (15). Thus, it can be seen as one way to cope with the stress that an individual feels from the environment, especially for middle school students whose peer influence is greater than their relationship with their parents (16).

Physical self-efficacy is defined as an individual’s level of confidence and ability to perform a specific task related to the body (17). In other words, feeling anxious and depressed in a crisis is a result of feeling lethargic because there is no way to cope with the situation. If control is possible in a crisis, anxiety and depression are reduced because of high self-efficacy (18). This physical self-efficacy is significant in predicting human behavior as a psychological factor of confidence concerning exercise behavior for the body (19).

Accordingly, various studies have been conducted on physical self-efficacy and exercise task performance. Hamilton et al. (20) found that active physical activity in adolescents is indirectly predicted by self-efficacy through intention, and this intervention was further controlled by the level of support from friends, demonstrating that friend support can partially buffer the lack of self-efficacy (20). Furthermore, among high school students who participated in the school sports association, physical self-efficacy and psychological well-being were reported to be related to ego resilience and psychological well-being, respectively (21). Therefore, participation in different physical activities during adolescence is an important factor in achieving a positive quality of life by improving physical self-efficacy (22).

Happiness through school life has a significantly positive effect on adolescents’ creativity and psychological energy (23). Happiness relates to the practical achievement of life goals and is a personal assessment of overall life satisfaction (24, 25). School happiness has a cognitive and emotional characteristic that evaluates students’ thoughts about themselves or the overall school environment, such as classes and relationships, based on what they experience in the learning process or human relationships (26). Happiness includes concepts such as subjective well-being, quality of life, and life satisfaction. According to previous studies, physical self-efficacy and relationships with parents, friends, and teachers were suggested as factors that affect happiness among students’ individual characteristics. Particularly, physical self-efficacy has a positive relationship with students’ school happiness and has been consistently reported in almost all preceding studies (27). Moreover, through the verification of happiness in school life, it is possible to positively control social deviance factors related to youth risk behavior (smoking, drugs, violence, drinking, social cheating, etc.,) and to conclude that sociality in school groups is doing well (28, 29).

The above analysis of the literature suggests that playfulness, as perceived by adolescents, promotes positive functions such as happy emotions, pleasurable experiences, motivation, creativity, relationships, and relaxation. In addition, playfulness has been shown to increase physical self-efficacy, which in turn improves adjustment to relationships with teachers and peers and adjustment to school life. Playfulness and physical self-efficacy were also strongly associated with education for happiness. Notably, these factors are closely related to the type, intensity, and frequency of physical activity participation during adolescence. Therefore, it may be important to investigate whether regular “0th-period PE class” for middle school students can positively predict the relationship between their playfulness, physical self-efficacy, and education for happiness. Specifically, there is a lack of research on the positive association between playfulness, physical self-efficacy, and education for happiness through participation in “0th-period PE class.” Recently, Korean society has been experiencing social problems such as bullying, violence and suicide among adolescents. In this situation, the positive association between playfulness, physical self-efficacy, and education for happiness may be valid in providing limited ways to cope with social problems. However, there is a lack of studies that have explored the relationship between the three variables. Thus, the purpose of this study is to explore the relationship between playfulness, physical self-efficacy and education for happiness as experienced by middle school students participating in “0th-period PE class.”

### 1.1. Hypotheses and research framework

**Figure 1:**
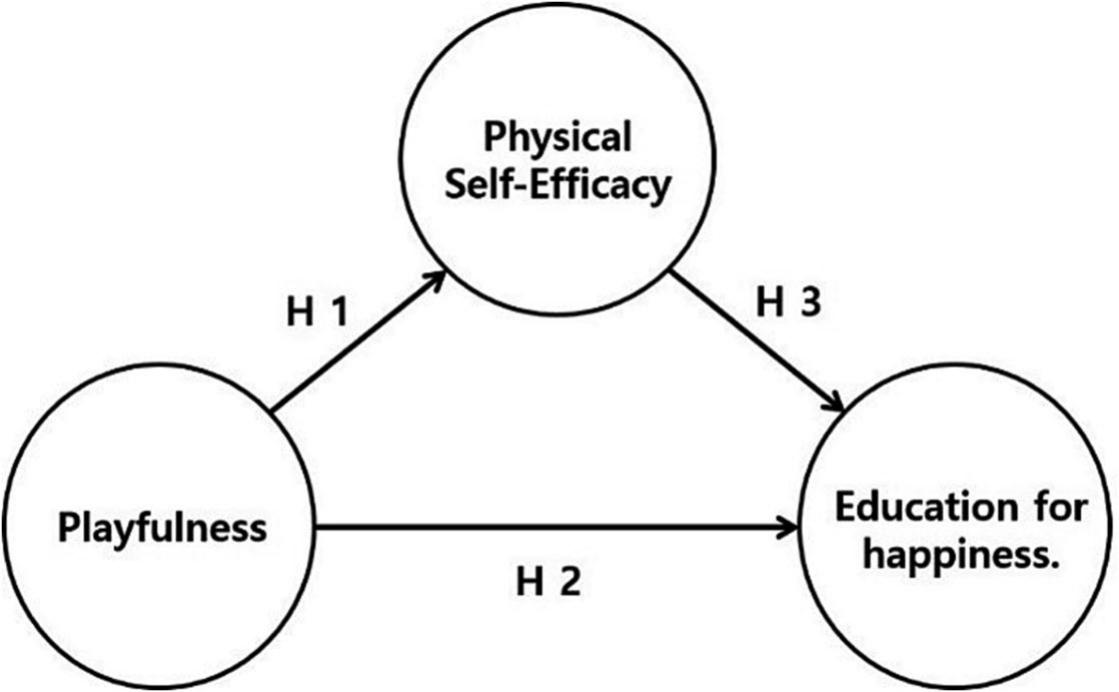
A conceptual model.

The hypotheses of this study were established based on the relationship between playfulness, physical self-efficacy and education for happiness. A conceptual model of the hypotheses is shown in Figure 1.

Figure 1 contains H1, which is the playfulness in the “0th-period PE class,” which positively affects towards physical self-efficacy. Secondly, H2 is the playfulness that positively affects education for happiness. Lastly, H3 is physical self-efficacy, which positively affects education for happiness.

## 2. Methods

### 2.1. Participants

In this study, middle school students located in Seoul and Gyeonggi-do, South Korea, were selected as sample groups. For sampling, 350 samples were extracted using a simple random sampling method among probability sampling methods. More specifically, given the characteristics of each region, Seoul was divided into four regions and one school was selected from each of the 28 cities in Gyeonggi-do. These were classified according to the legal administrative divisions in South Korea, and one school per division was randomly selected. Approximately 10–12 questionnaires were collected from each school. With the permission of the PE teachers at each school, the survey was conducted by two researchers using a face-to-face questionnaire with the students. Among the sampled survey data, 296 datasets were used for the final analysis, after eliminating 54 questionnaires that were judged to have been incorrectly indicated, or being incomplete. When removing the questionnaire, it was conducted by experts (statistical analysis experts and two PhD who majored in sociology of sport) other than researchers. The general characteristics of the study subjects are shown in (Table 1).

**Table 1:** General features of the study participants.

Section		Frequency	Percentage (%)
Sex	Female	170	57.4
	Male	126	42.6
Grade	First Grade	72	24.3
	Second Grade	192	64.9
	Third Grade	32	10.8

### 2.2. Measurements of key variables

To achieve the purpose of this study, we used structured questionnaires based on prior research and theory. The questionnaire comprises a total of 52 questions, including 2 items on demographic characteristics, 20 questions on playfulness, 10 questions on self-efficacy, and 20 questions on education for happiness. Playfulness was developed by Staempfli et al. (30). To confirm the factor structure of the Korean version of the ‘Adolescent Playfulness Scale’ and to test its reliability and conceptual validity, Kang (31) adapted the items of the ‘Adolescent Playfulness Scale’ (20 items) developed by Staempfli (30), conducted item analysis, and conducted exploratory and confirmatory factor analysis. Furthermore, the correlations between the sub-factors of the Korean version of the Youth Playfulness Scale and the openness and extraversion factors were examined, and the construct validity was tested. The Playfulness scale comprises 5 items: physical animation (4 questions), social engagement (4 questions), mental spontaneity (4 questions), emotional fluidity (4 questions), and humorous perspective (4 questions), for a total of 20 questions. The physical self-efficacy scale was measured by using the questions adopted and modified from the questionnaire developed by Ryckman et al. (17), and the questionnaire translated and used by Hong and Pyo (32) in Korean. The physical self-efficacy scale comprises two items: perceived physical ability (4 questions), and physical self-presentation confidence (6 questions), for a total of 10 questions. Education for happiness was measured using the questions adopted and modified from a previous study (33). It comprises five items: self-esteem (4 questions), thoughtfulness (4 questions), teacher-student relationship (4 questions), optimism (4 questions), and peer relationship (4 questions), comprising a total of 20 questions. The response of all of the above measurement tools comprised a 5-point Likert scale (from 1–strongly disagree to 5–strongly agree).

In this study, to examine the validity of the structured questionnaire, content validity verification of the questionnaire was conducted through consultation with three professors and two Ph.D. graduates who majored in the sociology of sport and sports pedagogy. To confirm the validity and internal consistency of the measurement tools, we conducted a confirmatory factor analysis by the Maximum likelihood method and a reliability analysis using Cronbach’s α. The outcomes are shown in Table 2.

**Table 2:** Confirmatory factor analysis and reliability of latent variables.

Factor	Latent variable		Measuring variables	B	β	S.E.	*t*	α
Playfulness	Physical Animation	→ → → →	a01 a02 a03 a04	1 1.069 1.430 1.299	0.576 0.654 0.825 0.799	0.180 0.201 0.186	5.94*** 7.10*** 6.98***	0.799
	Social Engagement	→ → → →	b01 b02 b03 b04	1 1.255 1.417 1.438	0.540 0.699 0.731 0.695	0.202 0.226 0.233	6.23*** 6.27*** 6.16***	0.766
	Mental Spontaneity	→ → → →	c01 c02 c03 c04	1 0.449 0.710 0.666	0.686 0.346 0.589 0.500	0.124 0.130 0.130	3.62*** 5.47*** 5.11***	0.946
	Emotional Fluidity	→ → → →	d01 d02 d03 d04	1 1.068 0.801 1.058	0.669 0.777 0.634 0.730	0.143 0.121 0.143	7.44*** 6.62*** 7.40***	0.805
	Humorous Perspective	→ → → →	e01 e02 e03 e04	1 1.178 1.288 1.045	0.726 0.782 0.861 0.745	0.127 0.126 0.121	9.31*** 10.19*** 8.66***	0.853
χ²: 262, df: 154, TLI: 0.903, CFI: 0.921, SRMR: 0.063, RMSEA: 0.068								
***p < 0.001								
Physical Self-Efficacy	Perceived Physical Ability Scale	→ → → →	a01 a02 a03 a04	1 −2.811 −4.243 −4.237	0.248 −0.629 −0.819 −0.847	1.054 1.520 1.499	−2.73** −2.79** −2.83**	0.736
	Physical Self-Presentation Confidence Scale	→ → → → → →	b01 b02 b03 b04 b05 b06	1 1.089 0.369 0.592 0.930 1.178	0.584 0.654 0.209 0.344 0.495 0.680	0.211 0.174 0.174 0.208 0.209	5.16*** 2.12* 3.41*** 4.47*** 5.64***	0.749
	χ²: 49.1 df: 34, TLI: 0.937, CFI: 0.952, SRMR: 0.062, RMSEA: 0.054								
	*p < 0.05, **p < 0.01, ***p < 0.001								
	Education for happiness	Self-esteem	→ → → →	a01 a02 a03 a04	1 0.848 1.128 1.386	0.718 0.508 0.695 0.798	0.142 0.141 0.150	5.95*** 7.99*** 9.25***	0.778
		Thoughtfulness	→ → → →	b01 b02 b03 b04	1 1.084 1.301 1.255	0.668 0.616 0.750 0.702	0.148 0.179 0.178	7.31*** 7.28*** 6.90***	0.780
Teacher relationship		→ → → →	c01 c02 c03 c04	1 1.110 1.648 1.577	0.563 0.429 0.761 0.643	0.265 0.238 0.269	4.18*** 6.92*** 5.85***	0.713
Optimism		→ → → →	d01 d02 d03 d04	1 1.046 1.060 1.135	0.727 0.697 0.779 0.783	0.265 0.238 0.269	4.18*** 6.92*** 5.85***	0.825
Peer relationship		→ → → →	e01 e02 e03 e04	1 1.226 0.993 0.986	0.616 0.664 0.616 0.655	0.153 0.165 0.154	7.99*** 6.00*** 6.39***	0.751
χ²: 256, df: 142, TLI: 0.902, CFI: 0.927, SRMR: 0.067, RMSEA: 0.073								
***p < 0.001								

### 2.3. Procedure and statistical analysis

To achieve the purpose of this study, the researcher visited the school in person and requested for the cooperation of the teacher in charge. Additionally, after being explained the purpose and method of filling out the questionnaire, the study participants were asked to complete the survey using the self-evaluation technique. If it was difficult for participants to respond to the survey face-to-face, it was conducted through a zoom program. The data collection in this study followed the following procedure: First, frequency analysis was conducted using the SPSS 21.0 program. Second, confirmatory factor analysis and reliability verification (Cronbach α) were conducted to verify the validity of the survey tool using the jamovi 1.2.27 program. Third, correlation analysis and standard multiple regression analysis were performed using the SPSS 21.0 program. The statistical significance probability of this study was set at 0.05.

## 3. Results

### 3.1. Correlation analysis among variables

Table 3 shows the correlation between each factor to determine the satisfaction of discriminant validity between each factor for factors identified as single dimensionality. Additionally, the correlation (r) between the relevant variables was −0.458 to 0.774, and it was shown that there was a partially significant correlation between the variables. Since the values of all correlation numbers did not exceed 0.80, discrimination was obtained based on the criteria in Kline (34). Furthermore, all variables appear smaller than 0.80—the criterion for multicollinearity between independent variables—indicating that there is no problem with multicollinearity (35).

**Table 3:** Correlation analysis among variables.

	1	2	3	4	5	6	7	8	9	10	11	12
1	1											
2	0.695**	1										
3	0.378**	0.519**	1									
4	0.387**	0.550**	0.459**	1								
5	0.565**	0.762**	0.575**	0.568**	1							
6	0.409**	0.368**	0.095	0.195*	0.312**	1						
7	0.143	0.187*	0.001	0.279**	0.129	0.460**	1					
8	0.428**	0.564**	0.526**	0.389**	0.502**	0.442**	0.362**	1				
9	0.276**	0.435**	0.496**	0.469**	0.337**	0.217**	0.318**	0.621**	1			
10	0.404**	0.473**	0.403**	0.434**	0.495**	0.393**	0.354**	0.699**	0.502**	1		
11	0.449**	0.560**	0.391**	0.483**	0.533**	0.430**	0.431**	0.760**	0.535**	0.763**	1	
12	0.578**	0.653**	0.454**	0.523**	0.636**	0.436**	0.352**	0.675**	0.514**	0.731**	0.774**	1

1. Physical animation, 2. Social engagement, 3. Mental spontaneity, 4. Emotional fluidity, 5. humorous perspective, 6. Perceived physical ability, 7. Physical self-presentation confidence, 8. Self-esteem, 9. Thoughtfulness, 10. Teacher relationship. 11. Optimism, 12. Peer relationship.

**p < 0.001.

### 3.2. Regression analysis results for playfulness on physical self-efficacy in “0th-period PE class”

Table 4 is the result of the influence of the playfulness of physical activity in the 0th-period of middle school students on their perceived physical ability and physical self-presentation confidence, which are sub-variables of physical self-efficacy.

**Table 4:** Regression analysis results for playfulness on physical self-efficacy.

Section	Perceived physical ability		Physical self-presentation confidence		VIF
	β	*t*	β	*t*	
Physical animation	0.295	2.817**	.033	.297	1.944
Social engagement	0.153	1.138	0.141	0.978	3.320
Mental spontaneity	−0.169	−1.800	−0.190	−1.917*	1.573
Emotional fluidity	0.005	0.050	0.314	3.314**	1.597
Humorous perspective	0.121	0.964	−0.066	−0.499	2.811
DW R² _adj_ F	1.850 R² = 0.172 F = 7.119***		2.060 R² = 0.078 F = 3.504**		

*p < 0.05; **p < 0.01; ***p < 0.001.

The regression model of playfulness and perceived physical ability was statistically significant (*p* < 0.001), the explanatory power of the regression model was found to be about 17.2% (R^2^_adj_ = 0.172), and the Durbin-Watson statistic was 1.850, showing a value close to 2, which was evaluated as no problem in the independence assumption of the residuals. The Variance Inflation Factor (VIF) was also found to be less than 10, indicating that there was no multicollinearity problem. Among the playfulness variables, physical animation (β = 0.295, *p* < 0.01) has confirmed a positive (+) influence relationship. This means that the higher the physical animation, the higher the perceived physical ability.

As a result of regression analysis between playfulness and physical self-presentation confidence, the regression model was statistically significant (p < 0.01), the explanatory power of the regression model was found to be about 7.8% (R^2^_adj_ = 0.078), and the Durbin-Watson statistic was 2.060, showing a value close to 2, which was evaluated as no problem with the independence of the residuals. Among the playfulness variables, emotional fluidity (β = 0.314) has (+) predictive effect on physical self-presentation confidence at the 0.1% level. Conversely, mental spontaneity (β = −0.190) has (−) predictive effect on physical self-presentation confidence at the 0.5% level.

### 3.3. Regression analysis results for playfulness on education for happiness in “0th-period PE class”

The results of the playfulness of middle school “0th-period PE class” on self-esteem, thoughtfulness, teacher relationship, optimism, and peer relationship, which are sub-variables of education for happiness, are shown in Table 5.

**Table 5:** Regression analysis results for playfulness on education for happiness.

Section	Self-esteem		Thoughtfulness		Teacher relationship		Optimism		Peer relationships		VIF
	β	*t*	β	*t*	β	*t*	β	*t*	β	*t*	
Physical animation	0.056	0.620	−0.050	−0.527	0.126	1.294	0.102	1.103	0.220	2.713**	1.944
Social engagement	0.331	2.788**	0.306	2.488**	0.078	0.610	0.237	1.963*	0.205	1.931*	3.320
Mental spontaneity	0.304	3.716***	0.361	4.271***	0.122	1.396	0.049	0.587	0.055	0.757	1.573
Emotional fluidity	0.032	0.393	0.291	3.415***	0.177	2.008*	0.206	2.456**	0.171	2.330*	1.597
Humorous perspective	0.024	0.220	−0.241	−2.132*	0.194	1.658	0.150	1.349	0.227	2.328*	2.811
DW R² _adj_ F	1.981 R² = 0.374 F = 18.596***		2.087 R² = 0.331 F = 15.555***		1.847 R² = 0.285 F = 12.730***		2.029 R² = 0.355 F = 17.164***		1.874 R² = 0.503 F = 30.742***		

*p < 0.05; **p < 0.01; ***p < 0.001.

As a result of regression analysis of playfulness and self-esteem, the regression model was statistically significant (*p* < 0.001), the explanatory power of the regression model was found to be about 37.4% (R^2^_adj_ = 0.374), and the Durbin-Watson statistic was 1.981 which was approximately 2 and evaluated as no problem in the independence assumption of the residuals. The Variance Inflation Factor (VIF) was also found to be less than 10, indicating that there was no multicollinearity issue. Among the playfulness variables, social engagement (β = 0.331) and mental spontaneity (β = 0.304) has confirmed a positive (+) influence relationship on the self-esteem at the 0.01 and 0.1% levels, respectively.

The explanatory power between playfulness and the sub-variable of education for happiness, thoughtfulness, in the middle school “0th-period PE class” was found to be 33.1% (R^2^_adj_ = 0.331). Among the playfulness variables, mental spontaneity (β = 0.361) and emotional fluidity (β = 0.291) had predictive (+) effect on the thoughtfulness scale at the 0.001% level and social engagement (β = 0.306) at the 0.01% level. Conversely, humorous perspective (β = −0.241) had predictive (−) effect on thoughtfulness at the 0.05% level.

In the findings of regression analysis between playfulness and teacher relationship, the regression model was statistically significant (*p* < 0.001), the explanatory power was found to be about 28.5% (R^2^_adj_ = 0.285), and the Durbin-Watson statistic was 1.847, which was close to 2, so it was evaluated that there was no problem with the independence of the residuals. Emotional fluidity (β = 0.177, *p* < 0.05) confirmed a positive (+) influence relationship in the teacher relationship as a result of testing the significance of the regression coefficient.

The explanatory power between playfulness and optimism was 35.5% (R^2^_adj_ = 0.355), and the DW statistic was 2.029. Among the playfulness variables, social engagement (β = 0.237) and emotional fluidity (β = 0.206) had a predictive (+) impact on optimism at the 0.05 and 0.01% level, respectively.

The explanatory power between playfulness and peer relationships was 50.3% (R^2^_adj_ = 0.503), and the DW statistic was 1.874. Among the playfulness variables, humorous perspective (β = 0.227, *p* < 0.05), physical animation (β = 0.220, *p* < 0.01), social engagement (β = 0.205, *p* < 0.05), and emotional fluidity (β = 0.171, *p* < 0.05) has confirmed a positive (+) influence relationship on the peer relationship.

### 3.4. Regression analysis results for physical self-efficacy on education for happiness in “0th-period PE class”

The results of the physical self-efficacy of middle school “0th-period PE class” on self-esteem, thoughtfulness, teacher relationship, optimism, and peer relationship, which are sub-variables of education for happiness, are illustrated in Table 6.

**Table 6:** Regression analysis results for physical self-efficacy on education for happiness.

Section	Self-esteem		Thoughtfulness		Teacher relationship		Optimism		Peer relationships		VIF
	β	*t*	β	*t*	β	*t*	β	*t*	β	*t*	
Perceived physical ability	0.350	4.251***	0.090	1.018	0.292	3.474***	0.294	3.634***	0.348	4.213***	1.269
Physical self-presentation confidence	0.201	2.442**	0.277	3.132**	0.219	2.610**	0.295	3.654***	0.192	2.319*	1.269
DW R² _adj_ F	2.172 R² = 0.216 F = 21.304***		2.186 R² = 0.095 F = 8.741***		1.930 R² = 0.181 F = 17.271***		2.072 R² = 0.243 F = 24.598***		1.868 R² = 0.209 F = 20.371***		

*p < 0.05; **p < 0.01; ***p < 0.001.

The regression model of physical self-efficacy and self-esteem was statistically significant (*p* < 0.001), the explanatory power of the regression model was found to be about 21.6% (R^2^_adj_ = 0.216), and the DW statistic was 2.172, showing a value close to 2, which was evaluated as no problem in the independence assumption of the residuals. The Variance Inflation Factor (VIF) was also found to be less than 10, indicating that there was no multicollinearity problem. Among the physical self-efficacy variables, perceived physical ability (β = 0.350, *p* < 0.001) and physical self-presentation confidence (β = 0.201, *p* < 0.01) have predictive (+) effect on self-esteem. This means that the higher the perceived physical ability and physical self-presentation confidence, the higher the self-esteem.

As a result of regression analysis of physical self-efficacy and thoughtfulness, the regression model was statistically significant (*p* < 0.001), the explanatory power of the regression model was found to be about 33.1% (R^2^_adj_ = 0.331), and the DW figure was 2.186 which was approximately 2 and evaluated as no problem in the independence assumption of the residuals. The Variance Inflation Factor (VIF) was no multicollinearity issue. Among the physical self-efficacy variables, physical self-presentation confidence (β = 0.277) had predictive (+) effect on the thoughtfulness scale at the 0.01% level.

The explanatory power between physical self-efficacy and the sub-variable of education for happiness, teacher relationship, in the middle school “0th-period PE class” was found to be 18.1% (R^2^_adj_ = 0.181). Moreover, the DW figure was 1.930. Among the physical self-efficacy variables, perceived physical ability (β = 0.292) and physical self-presentation confidence (β = 0.219) has confirmed a positive (+) influence relationship on the teacher relationship at the 0.001 and 0.01% levels, respectively.

The explanatory power between physical self-efficacy and optimism was 24.3% (R^2^adj = 0.243), and the DW was 2.072. Among the physical self-efficacy variables, physical self-expression confidence (β = 0.295, *p* < 0.001) and perceived physical ability (β = 0.294, *p* < 0.001) has confirmed a positive (+) influence relationship on the optimism.

In the findings of regression analysis between physical self-efficacy and peer relationship, the regression model was statistically significant (p < 0.001), the explanatory power was found to be about 20.9% (R^2^_adj_ = 0.209), and the DW was 1.868. Perceived physical ability (β = 0.348) and physical self-presentation confidence (β = 0.192) has confirmed a positive (+) influence relationship on the peer relationship at the 0.001 and 0.05% levels, respectively.

## 4. Discussion

This study sought to determine the relationships of playfulness experienced in early morning exercises in middle school students on physical self-efficacy and education for happiness. There was some evidence from overseas case studies as well as from the Korean education community of the positive and varied effects of morning exercise before classes (2, 6, 7, 36, 37), and it was intended to create an academic basis for establishing regular classes in schools soon. On the basis of the results of this study, we propose the following:

First, in the “0th-period PE class,” which has been operational in several middle schools in South Korea, the playfulness experienced by students had a predictive effect on physical self-efficacy. The implication of this finding is that playfulness in early morning exercise could be an effective means of increasing students’ physical self-efficacy. Results from a study of high school students in Korea also showed that morning exercise had a positive effect on the development of physical self-concept (38). Many scholars have tried to find out what internal and external factors affect the playfulness of infants, children, and adolescents, and how they are related to playfulness and development (39, 40). Since playfulness comprises physical, social, and cognitive spontaneity, expression of pleasure, and sense of humor (41), it is highly likely to feel pleasure and freedom through the playfulness factor and further transfer to positive human personality characteristics (42). For this reason, it is judged that the program of “0th-period PE class” needs to be planned focusing on the playfulness of students. Particularly, judging from the results of this study, it is quite significant to organize a program to maximize the positive effect of play on physical self-efficacy. This is because the quality and style of early morning exercise in which students participate is important (43). Furthermore, it is crucial to understand the psychological, emotional, and behavioral tendencies of students before organizing the program. This is because this process will not only have a productive effect on students’ acquisition of physical self-efficacy but can also be a sure means of improving their quality of life and invigorating them throughout their lives (44).

Second, we investigated the associations between playfulness and education for happiness and found some evidence of impact. According to previous studies, youths with excellent playfulness have exceptional creativity and problem-solving skills, are highly likely to perform outstandingly in academics, and are highly likely to have a smooth school life and high school happiness. This is because it is easy for students to acquire an internal disposition toward their environment, which exists in the inner dimension of a person through playfulness, which largely influences their choices and behavior (8). In other words, playfulness, a personal strategy that explains behavior, allows students to feel happy in various situations they face in school life. Ratey and Hagerman (45) introduced in the media the amazing effect of physical activity in the 0th-period by presenting students at Naperville High School in Illinois as an example. Additionally, it was emphasized that early morning exercise should be approached as a lifestyle logic rather than an educational aspect. More specifically, it was announced that it relieves students of their stress or anxiety and has a positive impact on emotional control. As shown in the findings of this study, “0th-period PE class” can be the basis for a major educational strategy that can increase students’ school happiness. Pekrun (46) proved that emotions affect adolescents’ learning, and stated that the development of emotions related to achievement is formed by individual factors and social environments. Since the emotion of achievement is defined as an emotion related to the results of success and failure of sports activities (46), “0th-period PE class” can affect achievement emotions and academic performance, and can be a basis for connecting to school happiness. According to a study conducted in Korea, high school students mentioned that early morning exercise has become a vital source of school life (8). It is in line with the results of this study. More importantly, the successful participation experience of early morning exercise has a positive effect on attitudes toward physical education classes (47). This can be of great academic significance that can lead to the institutional establishment of the “0th-period PE class” along with the results of this study.

Third, physical self-efficacy was found to have a predictive effect on education for happiness. The physical self-efficacy acquired by students in PE classes in the 0th-period can be inferred as a cause of increasing school happiness in various aspects. The physical self-efficacy scale, developed by Ryckman (17), has been widely used in exercise science. Particularly, Sonstroem (48) stated that physical activities such as exercise and dance increase an individual’s objective physical ability, resulting in a positive change in self-perception of physical ability, thereby increasing subjective physical ability—a secondary self-evaluation of physical performance. Ben-Eliyahu et al. (49) found in a study related to youth’s deep interests (sparks) that the most powerful spark is related to sports activities, and that it is deeply related to the positive developmental outcomes of adolescents. These changes are important for a healthy life. In addition, an individual’s perceived self-efficacy has a very great influence on their proper social life or school life. After all, strong self-efficacy induces a desire to achieve, reduces stress, and reduces vulnerability to negative emotions such as depression, thereby improving individual achievement and well-being (50). As shown in a study by Jeon et al. (7), students who participated in the “0th-period PE class” showed a positive perception of school life adaptation. Additionally, physical activity in the 0th-period has a positive effect on the physical strength and physical self-efficacy of middle school students and is in line with the results of the study that showed that it has a positive effect on learning attitudes (51). In this regard, the physical self-efficacy gained through early morning exercise may be an important factor in school satisfaction.

Notably, in Korea, although sports activities in the 0th-period are conducted through various programs, they are not legally mandated, and are mainly carried out according to the teachers’ will. Conversely, overseas, sports activities in the 0th-period are carried out based on legal grounds, schools and community infrastructure are organically linked, and education rights have been guaranteed (52). Therefore, besides the results of this study, we would like to suggest that the “0th-period PE class” comprising various activities, should be legally mandated in Korea.

Nevertheless, the results of this study have a limitation in that it is only indirectly supported, not a direct procedure for institutionalization of “0th-period PE class.” First, the current study focused on the relationships between variables analyzed through regression analysis, which limited the analysis of differences in demographic characteristics by gender and grade. If the study had focused on the relationships between variables based on gender or grade differences, the results may have been interpreted differently. Second, this study is limited to the personal psychological variables of playfulness, physical self-efficacy, and school happiness as perceived by middle school students. In other words, there are limitations in drawing connections between variables such as students’ academic performance and attitudes toward physical education. Therefore, future research should focus on the impact of various school physical education activities on students’ school life and social adjustment by examining the relationship between macro variables. Third, although there were no controls for gender, race, family characteristics, teacher characteristics, and school characteristics in the regression analysis, it is possible that these variables affect students’ attitudes toward physical education and academic performance.

## 5. Conclusion

The purpose of this study is to investigate the effect of middle school students’ playfulness in the “0th-period PE class” on their physical self-efficacy and education for happiness. Based on the analysis findings, the following conclusions were obtained: First, playfulness experienced through early morning exercise in middle school had a significant effect on physical self-efficacy. Second, playfulness had a significant effect on education for happiness. Third, physical self-efficacy had a significant effect on education for happiness.

Meanwhile, we would like to make the following suggestions for the parts that were not covered in this study and for active follow-up studies in the future. Physical activity in the 0th-period of middle school can increase satisfaction with school life by increasing physical perception in adolescence. In this respect, since early morning exercise is expected to have a positive effect on peer relationships, it is necessary to examine in depth the link between peer relationships and physical activity included in the 0th-period of physical activity in the future. Thus, it is expected to be used as basic data to prevent social problems such as school violence and bullying in Korean society.

## Data availability statement

The original contributions presented in the study are included in the article/supplementary material, further inquiries can be directed to the corresponding authors.

## Ethics statement

The studies involving humans were approved by Kangnam University Institutional Review Board. The studies were conducted in accordance with the local legislation and institutional requirements. Written informed consent for participation in this study was provided by the participants’ legal guardians/next of kin. Written informed consent was obtained from the minor(s)’ legal guardian/next of kin for the publication of any potentially identifiable images or data included in this article.

## Author contributions

BL and KJ: original draft preparation. WJ: data analysis. WJ and BL: critical review of the contents. KJ and WJ: data collection and critical review of the manuscript. KJ, WJ, and BL: supervision. All authors contributed to the article and approved the submitted version.

## Conflict of interest

The authors declare that the research was conducted in the absence of any commercial or financial relationships that could be construed as a potential conflict of interest.

## Publisher’s note

All claims expressed in this article are solely those of the authors and do not necessarily represent those of their affiliated organizations, or those of the publisher, the editors and the reviewers. Any product that may be evaluated in this article, or claim that may be made by its manufacturer, is not guaranteed or endorsed by the publisher.
